# Changes in primary healthcare providers’ attitudes and counseling behaviors related to dietary sodium reduction, DocStyles 2010 and 2015

**DOI:** 10.1371/journal.pone.0177693

**Published:** 2017-05-22

**Authors:** Zerleen S. Quader, Mary E. Cogswell, Jing Fang, Sallyann M. Coleman King, Robert K. Merritt

**Affiliations:** 1 Division for Heart Disease and Stroke Prevention, Centers for Disease Control and Prevention, Atlanta, Georgia, United States of America; 2 IHRC, Inc., Atlanta, Georgia, United States of America; Vanderbilt University, UNITED STATES

## Abstract

High blood pressure is a major risk factor for cardiovascular disease. The 2013 ACC/AHA Lifestyle Management Guideline recommends counseling pre-hypertensive and hypertensive patients to reduce sodium intake. Population sodium reduction efforts have been introduced in recent years, and dietary guidelines continued to emphasize sodium reduction in 2010 and 2015. The objective of this analysis was to determine changes in primary health care providers’ sodium-reduction attitudes and counseling between 2010 and 2015. Primary care internists, family/general practitioners, and nurse practitioners answered questions about sodium-related attitudes and counseling behaviors in DocStyles, a repeated cross-sectional web-based survey in the United States. Differences in responses between years were examined. In 2015, the majority (78%) of participants (n = 1,251) agreed that most of their patients should reduce sodium intake, and reported advising hypertensive (85%), and chronic kidney disease patients (71%), but not diabetic patients (48%) and African-American patients (43%) to consume less salt. Since 2010, the proportion of participants agreeing their patients should reduce sodium intake decreased while the proportion advising patients with these characteristics to consume less salt increased and the prevalence of specific types of advice declined. Changes in behaviors between surveys remained significant after adjusting for provider and practice characteristics. More providers are advising patients to consume less salt in 2015 compared to 2010; however, fewer agree their patients should reduce intake and counseling is not universally applied across patient groups at risk for hypertension. Further efforts and educational resources may be required to enable patient counseling about sodium reduction strategies.

## Introduction

Approximately 33% of U.S. adults have hypertension and another 31% have pre-hypertension, increasing their risk for cardiovascular diseases such as heart disease and stroke. Reducing sodium intake can lower blood pressure, with primary care provider counseling to reduce intake recommended by the American College of Cardiology/American Heart Association 2013 Lifestyle Management Guideline [1, 2]. Primary care providers’ advice plays an important role in influencing the lifestyle and health-related behaviors of patients [3]. Evidence suggests that the reported prevalence of taking action to reduce sodium intake is associated with reportedly receiving provider advice to do so. Eighty-three percent of adults who report receiving advice report taking action, compared with 44% of adults who do not report receiving advice [4]. However the proportion of adults living in U.S. who report receiving sodium or other diet-related lifestyle advice is only 20–35% [4–7].

While many report on patients’ receipt of provider advice regarding lifestyle modifications, fewer have examined such advice from the provider’s perspective and to our knowledge, no studies examine changes in advice over time. DocStyles, a web-based survey of health care providers, includes questions on providers’ opinions and counseling behaviors regarding sodium reduction. In 2010 the majority of primary healthcare providers agreed that most of their patients should reduce sodium intake [8]. With guidelines specifically addressing sodium reduction for hypertension prevention and management, as well national and state programs on sodium reduction underway, it is important to assess any changes in primary healthcare providers’ attitudes and behaviors regarding sodium intake. The objective of the present analysis is to determine how attitudes and counseling behaviors related to dietary sodium intake among primary healthcare providers have changed from 2010 to 2015.

## Materials and methods

### Data source

DocStyles is a web-based survey with a main sample of health care providers, including primary care physicians and nurse practitioners. The Centers for Disease Control and Prevention (CDC) licenses access to the data from the DocStyles surveys from Porter Novelli. Personal identifiers are not included in the datasets used for analyses, therefore this research was determined to be exempt from CDC IRB review. DocStyles was most recently conducted in 2015 and previously in 2012 and 2010. In 2010 and 2015 family and general practitioners, internists, and nurse practitioners were asked similar questions regarding sodium reduction. Each survey year, provider samples are drawn from a panel of medical practitioners in the United States. Sampling information and differences between the 2010 and 2015 surveys are described in Table 1. In both survey years participants were screened to include only those who practiced in the United States, actively saw patients, worked in an individual, group, or hospital practice, and who had been practicing for at least three years. Demographics of both years’ samples closely matched the American Medical Association’s master file proportions for age, gender, and region.

**Table 1:** Differences between 2010 and 2015 DocStyles survey^a^.

	2010	2015
Panel	Epocrates Honors Panel^b^	SERMO’s Global Medical Panel^c^
**Primary Care Physicians**^**d**^		
**Sample released**^e^	1,877	1,122
**Quota**	1,000	1,000
**# Complete (response rate)**^f^	1,000 (53.3%)	1,000 (89.1%)
**Nurse Practitioner**		
**Sample released**^e^	431	487
**Quota**	250	250
**# Complete (response rate)**^f^	254 (58.9%)	251 (51.5%)

^a^ Data provided by Porter Novelli (PN)

^b^ includes over 168,000 medical professionals in the U.S

^c^ includes over 330,000 medical professionals in the U.S. SERMO’s attempts to remove primary care physicians who consistently fail to respond to surveys.

^d^ includes family/general practitioners and internists

^e^ The number of providers who received the survey. Surveys are sent on a rolling basis until quotas are reached. In 2015, the survey timeframe was increased to allow for more reminders and quotas were met while releasing fewer surveys.

^f^ 2015 response rates provided by PN. 2010 response rates calculated as [# complete/sample released].

### Assessment of providers’ opinions and counseling behaviors regarding dietary sodium reduction

In 2010 and 2015, DocStyles contained 113 and 131 questions respectively, designed to provide insight into healthcare providers’ attitudes and counseling behaviors for various health issues. A sodium intake component was included in the survey, consisting of questions assessing health providers’ opinions and counseling behaviors related to reducing dietary sodium:

1. Most of my patients should reduce their dietary sodium intake (*strongly disagree*, *disagree*, *neither agree nor disagree*, *agree*, *strongly agree*)
2. Which of the following types of patients do you advise to consume less salt? Select all the apply (*pre-hypertensive patients*, *hypertensive patients*, *patients with chronic kidney disease (CKD)*, *Diabetic patients*, *Hispanic patients*, *African American patients*, *Asian patients*, *Adults older than 40 years old*, *all adults*, *none of these)*.
3. What specific advice do you provide your patients about how to consume less salt? Select all that apply (*read nutrition labels for sodium content*, *give examples of specific foods to avoid*, *remove salt shaker from the table*, *eat less processed food*, *cook with less sodium*, *other advice*, *do not provide specific advice)*
4. [Asked in 2015 only]What is your biggest barrier to discussing ways to reduce dietary sodium intake with hypertensive or pre-hypertensive adult patients? Select all that apply (*No major barriers*, *Not enough scientific evidence*, *Lack of resources for patient education*, *Patients have other immediate health issues*, *Patients are unlikely to comply*, *Lack of reimbursement*, *Lack of time)*

The survey also collected information on providers’ demographic and health characteristics, including age, race/ethnicity, sex, and self-reported height and weight, used to calculate body mass index (BMI). In 2010, responses to height and weight questions were complete, however in 2015 respondents 188 providers were missing height or weight data, and therefore missing BMI, in the current analysis. Questions about their practice consisted of the type of practitioner, their main work setting, years of practice, whether they had privileges at a teaching hospital, and the perceived financial situation (i.e. poor, lower middle class, affluent) of the majority of their patients.

### Statistical analysis

Differences in providers’ demographic, health, and practice characteristics, and responses to sodium questions between 2010 and 2015 were assessed using chi-square tests for categorical variables. Multiple logistic regression models were used to estimate unadjusted and adjusted odds ratios and 95% confidence intervals of giving advice to consume less salt for all patient types and for giving advice to patients to consume less salt in 2015 vs. in 2010. To examine whether differences between years in provider attitudes and behaviors were independent of differences in provider/practice characteristics, we adjusted for provider age and any variables that showed statistically significant differences between surveys. Participants with missing BMI were excluded from analyses. All analyses were conducted in 2016 using SAS software (version 9.3, SAS Institute, Inc., Cary, NC).

## Results

In 2010 and 2015, the majority of providers responding were male and non-Hispanic white, with a higher proportion of male providers and non-Hispanic Asian providers in 2015 vs. 2010 (Table 2). However, the proportion of non-Hispanic Asian nurse practitioners did not change between 2010 and 2015 (S1 Table). A majority of providers had normal BMI, with more normal-weight providers in 2015. Most respondents worked in group outpatient practices, and among family/general practitioners (FGPs), fewer respondents worked in inpatient practice in 2015 (S1 Table).

**Table 2:** Demographic, health, and practice characteristics of primary healthcare providers, DocStyles 2010 and 2015.

	2010 (n = 1,254)	2015 (n = 1,251)	p-value^a^
**Specialty, %**			
Family/General Practitioner	43.0	37.2	0.004
Internist	36.8	42.8	
Nurse Practitioner	20.3	20.1	
**Age, y, %**			
< 45	50.9	49.2	0.41
≥ 45	49.1	50.8	
**Gender, % male**	56.2	62.0	0.003
**Race/ethnicity, %**			
Non-Hispanic white	74.4	62.2	< .0001
Non-Hispanic black	3.4	2.5	
Hispanic	3.4	4.0	
Non-Hispanic Asian	15.1	24.3	
Other^b^	3.8	7.0	
**Body mass index**^c^**,%**			
< 25.0	48.9	56.0	0.001
25.0–29.9	36.8	33.5	
> 30	14.3	10.5	
**Years practicing medicine, %**			
< 10	32.4	29.9	0.23
10–19.9	41.0	40.7	
≥ 20	26.6	29.4	
**Main work setting**^d^**, %**			
Individual outpatient practice	17.2	19.3	0.003
Group outpatient practice	61.0	64.2	
Inpatient practice	21.8	16.5	
**Working at a teaching hospital, %**	42.2	46.8	0.02
**Financial situation of majority of patients**^e^**, %**			
Poor	5.4	7.0	< .0001
Lower middle	15.2	23.3	
Middle	42.2	34.5	
Upper middle	32.9	26.1	
Affluent	4.3	9.3	

^a^p-value based on chi-square tests for differences in the proportion responding across year

^b^includes Native Hawaiian or other Pacific Islander, American Indian or Alaska Native, or multi-racial respondents

^c^For adults aged ≥ 20, normal weight = BMI < 25 kg/m^2^; overweight = 25 kg/m^2^ ≤ BMI < 30 kg/m^2^; obese = BMI ≥ 30 kg/m^2^; in 2015 missing n = 188

^d^2015 answer choices; 2010 answer choices: individual practice, group practice, hospital or clinic

^e^2015 answer choices: Poor (< $25,000), lower middle ($25,000 - $49,000), middle ($50,000 - $99,000), upper middle ($100,000 - $249,000), upper (≥$250,000). 2010 answer choices: very poor-poor; poor—lower middle class; lower middle class—middle class; middle class—upper middle class; upper middle class–affluent

In both survey years, the majority (86%) of respondents agreed (agree or strongly agree) with the statement that most of their patients should reduce their sodium intake, and the majority (65% or more) also reported advising pre-hypertensive, hypertensive, or chronic kidney disease patients to consume less salt (Table 3). Across provider types, 51% of internists and 54% of nurse practitioners in 2015 reported advising diabetic patients to consume less salt, compared with 43% of FGPs (S2 Table). Compared with 2010, in 2015 the proportion of providers who reported advising pre-hypertensive patients to consume less salt did not significantly change, but the proportion who reported advising other patients increased (Table 3). In addition, higher proportions of all provider types reported advising hypertensive patients and African American patients to consume less salt, and internists reported advising CKD and diabetic patients to consume less salt (S2 Table).

**Table 3:** Primary healthcare providers' attitudes and counseling related to dietary sodium reduction, DocStyles 2010 and 2015.

	2010 (n = 1,254)	2015 (n = 1,251)	p-value^a^
**Agreement with statement "Most of my patients should reduce their sodium intake." (%)**^**b**^			
Strongly disagree	0.3	1.4	
Disagree	2.6	5.3	
Neither agree nor disagree	11.1	15.8	< .0001
Agree	55.4	49.1	
Strongly agree	30.6	28.4	
**Which of the following types of patients do you advise to consume less salt? (%)**			
Pre-hypertensive patients	65.7	68.8	0.11
Hypertensive patients	74.2	84.2	< .0001
Chronic kidney disease patients	65.0	71.1	0.001
Diabetic patients	43.5	48.4	0.016
Hispanic patients	18.4	23.4	0.002
African American patients	33.9	43.3	< .0001
American Indian patients	14.3	20.5	< .0001
Asian patients	12.5	18.3	< .0001
Adults older than 40 years old	19.9	25.3	0.001
All adults^c^	—	31.3	—
**What specific advice do you provide patients to consume less salt? (%)**			
Read nutrition labels for sodium content	86.8	74.8	< .0001
Give examples of specific foods to avoid	77.9	65.2	< .0001
Remove the salt shaker from the table	68.9	56.8	< .0001
Eat less processed food	86.8	78.2	< .0001
Cook with less sodium	73.1	66.5	0.0004
Other advice	7.9	6.1	0.07
Do not provide advice	1.5	3.5	0.001

^a^p-value based on chi-square tests for differences in the proportion responding across year

^b^based on Mann Whitney U test

^c^2010 “all adults” answer choice was exclusive only and 2015 was “select all.” Therefore, results are not comparable.

The most commonly reported salt-reduction advice was to “eat less processed food,” followed by “read nutrition labels for sodium content” (Table 3). Compared with 2010, in 2015 the proportion of FGPs and internists providing specific advice was significantly lower for all types of salt-reduction advice. In contrast, a lower proportion of nurse practitioners reported advice on “specific foods to avoid” and removing the salt shaker from the table, but not other types of advice (S2 Table).

**Fig 1:**
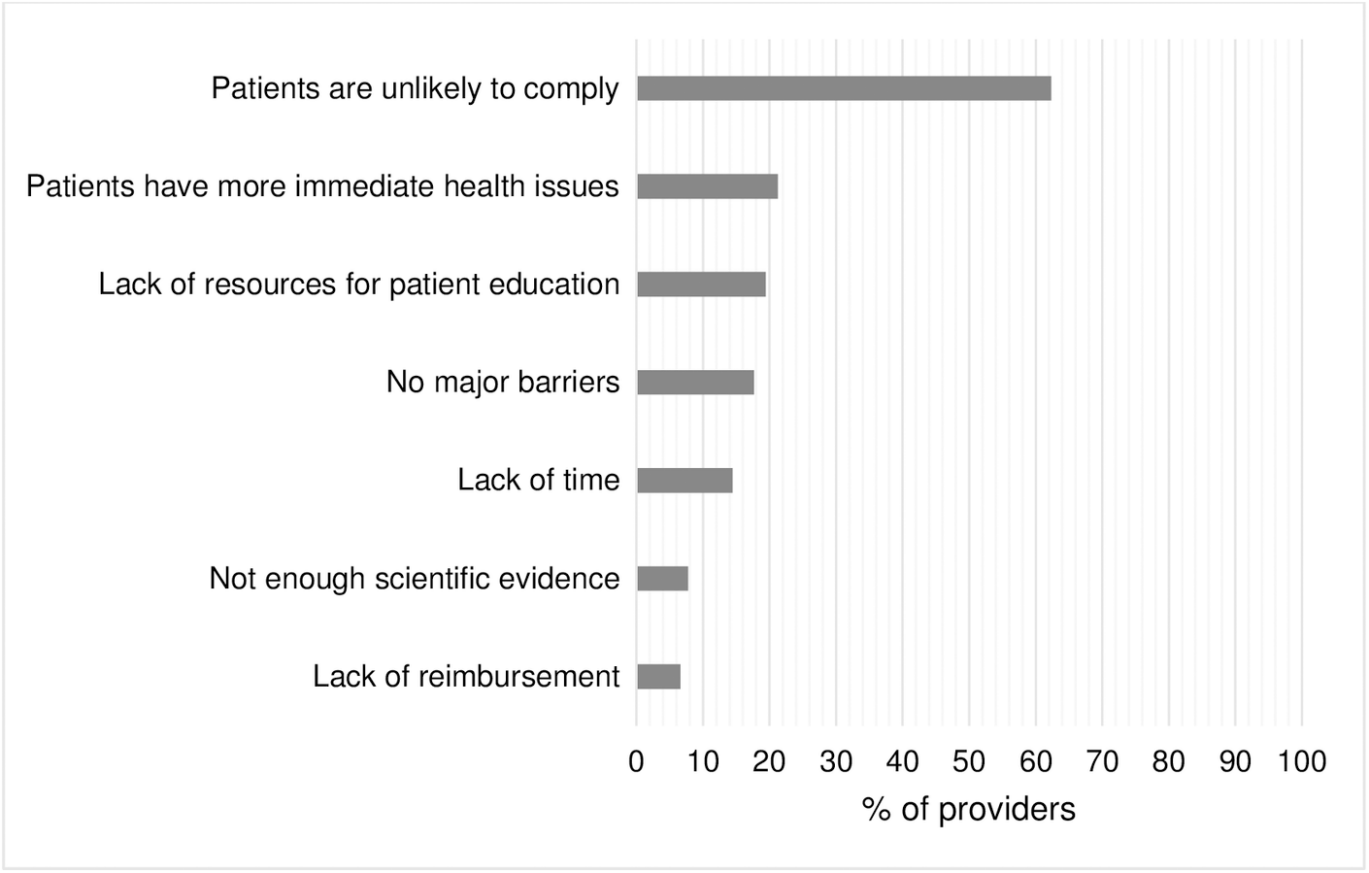
"What is your biggest barrier to reduce dietary sodium intake with hypertensive or pre-hypertensive patients? Select all that apply"—DocStyles 2015.

In 2015, the most frequently reported barrier to reducing dietary sodium intake with hypertensive or pre-hypertensive patients was that “patients are unlikely to comply.” There were no significant differences by provider type for any reported barriers, except for “not enough scientific evidence.” Ten percent of FGPs and 8% of internists reported “not enough scientific evidence” as a barrier, compared with 4% of nurse practitioners (Fig 1).

Most differences between 2010 and 2015 in primary care providers’ sodium reduction attitudes and counseling remained significant after adjustment for demographic, health, and practice characteristics. One exception was that the temporal difference in the odds of advising diabetic patients to consume less salt in 2015 vs. 2010 was no longer significant (Table 4).

**Table 4:** Unadjusted and adjusted odds ratios and 95% confidence interval of primary healthcare providers' attitudes and counseling related to dietary sodium reduction in 2015 versus 2010, DocStyles^a^.

	OR^b^	95% CI	AOR^c^	95% CI
**Agreement with statement "Most of my patients should reduce their sodium intake."**^d^	0.56	(0.45–0.69)	0.57	(0.45–0.71)
**Which of the following types of patients do you advise to consume less salt?**				
Pre-hypertensive patients	1.15	(0.97–1.36)	1.27	(1.06–1.52)
Hypertensive patients	1.85	(1.52–2.26)	2.20	(1.76–2.74)
Chronic kidney disease patients	1.32	(1.12–1.57)	1.46	(1.21–1.75)
Diabetic patients	1.21	(1.04–1.42)	1.18	(1.00–1.41)
Hispanic patients	1.35	(1.12–1.64)	1.35	(1.09–1.66)
African American patients	1.49	(1.27–1.75)	1.56	(1.31–1.87)
American Indian patients	1.54	(1.25–1.91)	1.50	(1.19–1.88)
Asian patients	1.57	(1.26–1.95)	1.49	(1.17–1.89)
Adults older than 40 years old	1.36	(1.12–1.64)	1.44	(1.17–1.760
**What specific advice do you provide patients to consume less salt?**				
Read nutrition labels for sodium content	0.45	(0.37–0.56)	0.51	(0.41–0.64)
Give examples of specific foods to avoid	0.53	(0.45–0.64)	0.55	(0.45–0.66)
Remove the salt shaker from the table	0.59	(0.50–0.70)	0.65	(0.54–0.78)
Eat less processed food	0.55	(0.44–0.68)	0.60	(0.48–0.76)
Cook with less sodium	0.73	(0.62–0.87)	0.71	(0.59–0.86)

^a^2010 used as referent group

^b^Unadjusted odds ratio

^c^Adjusted for age, provider specialty, sex, race/ethnicity, BMI category, work setting, privileges at a teaching hospital, and financial situation of most of patients; missing n = 188 without BMI data.

^d^Agree or strongly agree

## Discussion

In 2015, the majority of healthcare providers agreed that most of their patients should reduce their sodium intake and reported advising patients to consume less salt, which is consistent with results from 2010 and current recommendations for hypertension control [1]. The majority of healthcare providers’ reported advising pre-hypertensive, hypertensive, and chronic kidney disease patients to reduce salt consumption. Previous studies indicate that subgroups that are at higher risk for cardiovascular conditions are more likely to report receiving advice from their healthcare professional about lifestyle changes [5, 9]. In the present analysis, reported advice to consume less salt has increased since 2010 for diabetic patients among internists and nurse practitioners only, and African American patients among all provider types. However, a majority of providers overall still do not report advising these populations to reduce sodium intake. This is important because African Americans have higher rates of hypertension than non-Hispanic whites and Hispanics, along with higher rates of uncontrolled hypertension, and patients with diabetes have a higher prevalence of hypertension compared to non-diabetic patients [10]. However, while evidence suggests that patients with a greater number of chronic conditions are more likely to report receiving advice regarding lifestyle change, it is possible that this advice is not necessarily focused on sodium reduction [5–7]. The annual number of office visits among African Americans is lower than for whites (2 v 3 per year), so more emphasis may be placed on other patient needs [11]. Furthermore, in the present analysis, “patients having more immediate health issues” was the second most reported barrier to sodium reduction, which could be true for diabetic patients, whose nutrition advice may focus on other dietary components important for diabetes management [12].

The *2015–2020 Dietary Guidelines for Americans* recommend that all adults reduce sodium intake to 2,300 mg/day, and suggest that adults with prehypertension and hypertension would benefit from a further reduction of sodium to 1,500 mg/day, consistent with ACC/AHA recommendations [13]. However, average intake among US adults remains high, at 3,592 mg/day [14]. The Institute of Medicine’s report on strategies to reduce sodium intake highlights the need for population-wide sodium reduction, and initiatives aimed at this are proposed or underway [15, 16]. As the United States moves forward with efforts to meet *Healthy People 2020* objectives to reduce population sodium consumption, it is important that advice provided by healthcare providers aligns with current recommendations, particularly for those with or at risk for high blood pressure and cardiovascular disease.

Provider advice can play in important role in patient care, particularly for encouraging lifestyle or behavior changes [17, 18]. Recent data indicate that patients who report receiving advice from their physician to reduce sodium intake are significantly more likely to report action to reduce sodium intake independent of sociodemographic and health characteristics [4]. The majority of individuals with diabetes and chronic kidney disease already report that they are watching or reducing their sodium intake and reported action was even higher among those that received advice [4]. Receiving advice from a healthcare provider could provide additional motivation to reduce sodium intake. However, evidence is mixed on whether receiving lifestyle advice translates into an actual change in diet or health outcomes. A systematic review of trials examining the effect of dietary advice, found that advice resulted in reduced sodium intake along with other dietary improvements [19]. However, an analysis of hypertensive adults from a nationally representative survey found no significant difference in sodium intake between those that reported taking action to reduce sodium after being given advice to do so and those that did not report taking action [20]. It is possible that more intensive counseling is effective at changing behavior, however even with considerable effort, the sodium density of the U.S. food supply may make it difficult to reduce consumption [21].

Commercially packaged and restaurant foods are estimated to contribute 75% of sodium intake in the US population, while approximately 11% is from discretionary salt [22, 23]. The current analysis indicates that providers’ recommendations align with this information, as the most commonly reported advice for reducing salt intake is to “limit processed foods” and “read nutrition labels for sodium content.” However, the odds of giving advice decreased from 2010 to 2015 for all types of advice. Difficulty in actually reducing sodium intake may explain this and also why providers reported noncompliance as the major barrier to sodium reduction, which has been reported in a previous study of health providers [24]. Hypertensive patients have also reported difficulty adhering to a low-sodium diet as a barrier to controlling blood pressure [25].

The current analysis also indicated differences in reported advice given by provider type (S2 Table). Between 2010 and 2015, a greater proportion of nurse practitioners reported advising all patient types, including diabetic patients and African American patients, to consume less salt compared to primary care physicians. Analysis of outpatient data from the National Hospital Ambulatory Medical Care Survey from 2005–2009 found that health education was not routinely provided to patients with chronic conditions, however nurse practitioners provided health education more regularly than physicians [26]. Potential reasons may be differences in training, patients may feel more comfortable discussing behaviors with them, or differences in demands that may allow nurse practitioners to be able to spend more time on individual patient consultations [27]. However, time and reimbursement were not frequently reported as barriers by any provider types. Additionally, more nurse practitioners in this sample were women than were other provider types. Evidence suggests that female healthcare providers are more likely to provide health behavior counseling and preventive services than their male counterparts, which may also partially explain these results [28, 29]. However in the current analysis, providers’ characteristics, including gender, though different in 2015 compared to 2010, did not have a large impact on most of the findings after adjustment.

Some limitations of this analysis should be addressed. First, results of this survey are dependent on providers’ self-reported behavior, which could differ from providers’ actual actions, especially if providers attempt to give a more socially-desirable response. Second, because the DocStyles survey is voluntary, these results may be biased if providers who are more concerned about patient care or lifestyle modification are more likely to respond to the survey. Third, between 2010 and 2015, there were differences in the surveys and the underlying panels samples were drawn from, which prevented some comparisons between years, led to missing BMI data on some providers, and could have led to more providers who are concerned with lifestyle modifications or patient care in 2015 than in 2010. Fourth, the survey used the term “sodium” in questions aimed at understanding providers’ opinions and “salt” in the questions aimed to determine providers’ advice related to reducing dietary sodium intake. Although similar, the two terms may have been interpreted differently. Lastly, DocStyles is not nationally representative. However, it is a large national survey with current information on participating healthcare providers and age and gender distributions closely correspond with those of physician members of the American Medical Association.

Overall, provider advice regarding reducing sodium consumption continues to align with American Heart Association/American College of Cardiology recommendations for hypertension control [1]. The majority of providers continue to agree that their patients should reduce sodium intake and more providers advised their patients to consume less salt in 2015 than in 2010. However, in 2015 a minority of providers recommend sodium reduction for all adults, despite high levels of intake in the general population, and there is room for improvement in recommending diabetic patients and African American patients to consume less salt, as they are at higher than average risk for hypertension. Further efforts may be required to enable providers to counsel their patients about salt reduction.

## Supporting information

S1 Table Demographic, health and practice characteristics of primary healthcare providers, by provider type DocStyles 2010 and 2015. (DOCX)

S2 Table Primary healthcare providers' attitudes and counseling related to dietary sodium reduction, by provider type, DocStyles 2010 and 2015. (DOCX)

## References

[1] Eckel RH, Jakicic JM, Ard JD, de Jesus JM, Houston Miller N, Hubbard VS, et al 2013 AHA/ACC guideline on lifestyle management to reduce cardiovascular risk: a report of the American College of Cardiology/American Heart Association Task Force on Practice Guidelines. J Am Coll Cardiol. 2014 7;63(25 Pt B):2960–84. 24239922 10.1016/j.jacc.2013.11.003

[2] Mozaffarian D, Benjamin EJ, Go AS, Arnett DK, Blaha MJ, Cushman M, et al on behalf of the American Heart Association Statistics Committee and Stroke Statistics Subcommittee. Heart disease and stroke statistics– 2016 update: report from the American Heart Association. Circulation. 2016; 133:000–000. 10.1161/CIR.0000000000000350 26673558

[3] Kreuter MW, Chheda SG, Bull FC. How does physician advice influence patient behavior? Evidence for a priming effect. Arch Fam Med. 2000;9(5):426–33. 10810947 10.1001/archfami.9.5.426

[4] Jackson SL, Coleman King SM, Park S, Fang J, Odom EC, Cogswell ME. Health professional advice and adult action to reduce sodium intake. Am J Prev Med. 2016 1;50(1):30–9. doi: 10.1016/j.amepre.2015.04.034 26163171 10.1016/j.amepre.2015.04.034 PMC5082829

[5] Corsino L, Svetkey LP, Ayotte BJ, Bosworth HB. Patient characteristics associated with receipt of lifestyle behavior advice. N C Med J. 2009 9;70(5):391–8. 19999515 PMC3333794

[6] Honda K. Factors underlying variation in receipt of physician advice on diet and exercise: applications of the behavioral model of health care utilization. Am J Health Promot. 2004 5;18(5):370–7. 15163138 10.4278/0890-1171-18.5.370

[7] Sinclair J, Lawson B, Burge F. Which patients receive advice on diet and exercise? Do certain characteristics affect whether they receive such advice? Can Fam Physician. 2008 3;54(3):404–12. 18337535 PMC2278358

[8] Fang J, Cogswell ME, Keenan NL, Merritt RK. Primary health care providers' attitudes and counseling behaviors related to dietary sodium reduction. Arch Intern Med. 2012 1;172(1):76–8. doi: 10.1001/archinternmed.2011.620 22232154 10.1001/archinternmed.2011.620 PMC4580130

[9] Lopez L, Cook EF, Horng MS, Hicks LS. Lifestyle modification counseling for hypertensive patients: results from the National Health and Nutrition Examination Survey 1999–2004. Am J Hypertens. 2009 3; 22(3):325 doi: 10.1038/ajh.2008.348 19096366 10.1038/ajh.2008.348

[10] Centers for Disease Control and Prevention. Prevalence of Hypertension and Controlled Hypertension—United States, 2007–2010. MMWR Morb Mortal Wkly Rep. 2013 11; 62(03):144–48.

[11] Ambulatory and Hospital Care Statistics Branch. National Ambulatory Medical Care Survey: 2012 State and National Summary Tables. [Internet]. Atlanta, (GA): Centers for Disease Control and Prevention and National Center for Health Statistics[updated 2016 Jul 26; cited 2016 Jul 27]. Available from: http://www.cdc.gov/nchs/ahcd/web_tables.htm

[12] Evert AB, Boucher JL, Cypress M, Dunbar SA, Franz MJ, Mayer-Davis EJ, et al Nutrition therapy recommendations for the management of adults with diabetes. Diabetes Care. 2014 1;37(Supplement 1):S120–43. 24357208 10.2337/dc14-S120

[13] U.S. Department of Agriculture and U.S. Department of Health and Human Services. 2015–2020 Dietary Guidelines for Americans. 8th ed. Washington DC. 2015.

[14] Agricultural Research Service, U.S. Department of Agriculture. WWEIA Data Tables: 2011–2012. [Internet]. Beltsville (MD): U.S. Department of Agriculture; [updated 2015 Feb 11; cited 2016 Jul 27]. Available from: http://www.ars.usda.gov/Services/docs.htm?docid=18349.

[15] Institute of Medicine. Strategies to Reduce Sodium Intake in the United States. Washington DC: National Academies Press, 2010.

[16] NYC Department of Health and Mental Hygiene. Sodium Initiative. [Internet]. New York City (NY): NYC Department of Health and Mental Hygiene [updated 2016; cited 2016 Jul 27]. Available from: https://www1.nyc.gov/site/doh/health/health-topics/national-salt-reduction-initiative.page.

[17] Viera AJ, Kshirsagar AV, Hinderliter AL. Lifestyle modifications to lower or control high blood pressure: is advice associated with action? The Behavioral Risk Factor Surveillance Survey. J Clinical Hypertens (Greenwich). 2008 2;10(2):105–11. 18256575 10.1111/j.1751-7176.2008.07577.x PMC8109971

[18] Valderrama AL, Tong X, Ayala C, Keenan NL. Prevalence of self-reported hypertension, advice received from health care professionals, and actions taken to reduce blood pressure among US adults—HealthStyles, 2008. J Clinical Hypertens (Greenwich). 2010 10;12(10):784–92. 21029341 10.1111/j.1751-7176.2010.00323.x PMC8673196

[19] Rees K, Dyakova M, Wilson N, Ward K, Thorogood M, Brunner E. Dietary advice for reducing cardiovascular risk. Cochrane Database Syst Rev. 2013(12). 10.1002/14651858.CD002128.pub5 PMC9993221 24318424

[20] Ayala C, Gillespie C, Cogswell M, Keenan NL, Merritt R. Sodium consumption among hypertensive adults advised to reduce their intake: National Health and Nutrition Examination Survey, 1999–2004. J Clinical Hypertens (Greenwich) 2012 7;14(7):447–54. 22747617 10.1111/j.1751-7176.2012.00632.x PMC6247783

[21] Adler AJ, Taylor F, Martin N, Gottlieb S, Taylor RS, Ebrahim S. Reduced dietary salt for the prevention of cardiovascular disease. Cochrane Database Syst Rev. 2014(12). 10.1002/14651858.CD009217.pub3 PMC6483405 25519688

[22] Centers for Disease Control and Prevention. Vital signs: food categories contributing the most to sodium consumption—United States, 2007–2008. MMWR Morb Mortal Wkly Rep 2012 2;61(5):92–8. 22318472

[23] Mattes RD, Donnelly D. Relative contributions of dietary sodium sources. J Am Coll Nutr. 1991;10(4):383–93. 1910064 10.1080/07315724.1991.10718167

[24] Wexler R, Elton T, Taylor CA, Pleister A, Feldman D. Physician reported perception in the treatment of high blood pressure does not correspond to practice. BMC Fam Pract. 2009 4;10(23): [5 p]. 10.1186/1471-2296-10-23 PMC2675522 19341474

[25] Donahue KE, Vu MB, Halladay JR, Miller C, Garcia BA, Cummings DM, et al Patient and practice perspectives on strategies for controlling blood pressure, North Carolina, 2010–2012. Prev Chronic Dis. 2014 4 24;11:E69 doi: 10.5888/pcd11.130157 24762533 10.5888/pcd11.130157 PMC4008946

[26] Ritsema TS, Bingenheimer JB, Scholting P, Cawley JF. Differences in the delivery of health education to patients with chronic disease by provider type, 2005–2009. Prev Chronic Dis. 2014 3 6;11:E33 doi: 10.5888/pcd11.130175 24602587 10.5888/pcd11.130175 PMC3944949

[27] Laurant M, Reeves D, Hermens R, Braspenning J, Grol R, Sibbald B. Substitution of doctors by nurses in primary care. Cochrane Database Syst Rev. 2005 4 20;(2). 10.1002/14651858.CD001271.pub2 15846614

[28] Tabenkin H, Eaton C, Roberts M, Parker D, McMurray J, Borkan J. Differences in cardiovascular disease risk factor management in primary care by sex of physician and patient. Ann Fam Med. 2010 1;8(1):25–32. doi: 10.1370/afm.1071 20065275 10.1370/afm.1071 PMC2807384

[29] Henderson JT, Weisman CS. Physician gender effects on preventive screening and counseling: an analysis of male and female patients' health care experiences. Med Care. 2001;39(12):1281–92. 11717570 10.1097/00005650-200112000-00004

