# Supplementary material for: Changes in primary healthcare providers’ attitudes and counseling behaviors related to dietary sodium reduction, DocStyles 2010 and 2015
Source: PLoS One. 2017 May 22;12(5):e0177693. doi: 10.1371/journal.pone.0177693 (PMC5439686; doi:10.1371/journal.pone.0177693)
Supplement: S1 Table — (DOCX) [file pone.0177693.s001.docx]

| **S1 Table: Demographic, health, and practice characteristics of primary healthcare providers, by provider type DocStyles 2010 and 2015** | | | | | | | | | |
| --- | --- | --- | --- | --- | --- | --- | --- | --- | --- |
|  | **Family/general practitioner** | | | **Internist** | | | **Nurse practitioner** | | |
|  | **2010** | **2015** | **P-value^a^** | **2010** | **2015** | **P-value^a^** | **2010** | **2015** | **P-value^a^** |
| **Age, y, %** |  |  |  |  |  |  |  |  |  |
| < 45 | 50.3 | 51.4 | 0.7 | 55.5 | 49.0 | 0.04 | 43.7 | 45.8 | 0.6 |
| ≥ 45 | 49.7 | 48.6 |  | 44.5 | 51.0 |  | 56.3 | 54.2 |  |
| **Gender, % male** | 66.4 | 70.3 | 0.2 | 70.9 | 77.8 | 0.01 | 7.9 | 13.2 | 0.05 |
| **Race, %** |  |  |  |  |  |  |  |  |  |
| Non-Hispanic white | 76.8 | 60.9 | <.0001 | 62.0 | 52.3 | 0.002 | 91.7 | 85.7 | 0.18 |
| Non-Hispanic black | 3.3 | 2.4 |  | 4.3 | 1.5 |  | 2.0 | 4.8 |  |
| Hispanic | 4.1 | 5.4 |  | 2.8 | 3.4 |  | 2.8 | 2.8 |  |
| Non-Hispanic Asian | 12.2 | 25.2 |  | 25.6 | 33.5 |  | 1.9 | 3.2 |  |
| Other | 3.5 | 6.2 |  | 5.2 | 9.3 |  | 1.6 | 3.6 |  |
| **Body mass index^b^,%** |  |  |  |  |  |  |  |  |  |
| < 25.0 | 42.7 | 53.3 | 0.001 | 56.4 | 56.1 | 0.97 | 48.4 | 60.2 | 0.03 |
| 25.0 - 29.9 | 41.4 | 37.1 |  | 34.5 | 34.4 |  | 31.5 | 25.2 |  |
| ≥ 30 | 15.9 | 9.6 |  | 9.1 | 9.5 |  | 20.1 | 14.2 |  |
| **Years practicing medicine, %** |  |  |  |  |  |  |  |  |  |
| < 10 | 29.9 | 26.7 | 0.4 | 34.5 | 29.0 | 0.17 | 33.9 | 37.8 | 0.0002 |
| 10—20 | 39.0 | 42.8 |  | 37.3 | 41.1 |  | 51.9 | 35.9 |  |
| ≥ 20 | 31.2 | 30.5 |  | 28.2 | 29.9 |  | 14.2 | 26.3 |  |
| **Main work setting**^c^**, %** |  |  |  |  |  |  |  |  |  |
| Individual outpatient practice | 21.2 | 22.4 | <.0001 | 13.9 | 17.0 | 0.01 | 15.0 | 18.7 | 0.07 |
| Group outpatient practice | 65.1 | 75.0 |  | 53.6 | 58.7 |  | 65.7 | 55.8 |  |
| Inpatient practice | 13.7 | 2.6 |  | 32.5 | 24.3 |  | 19.3 | 25.5 |  |
| **Working at a teaching hospital, %** | 36.4 | 38.5 | 0.5 | 57.3 | 58.7 | 0.7 | 27.2 | 37.1 | 0.02 |
| **Financial situation of majority of patients**^d^**, %** |  |  |  |  |  |  |  |  |  |
| Poor | 5.0 | 5.6 | <.0001 | 4.8 | 6.4 | <.0001 | 7.5 | 10.8 | 0.06 |
| Lower middle | 14.8 | 25.2 |  | 13.9 | 2.6 |  | 18.1 | 25.5 |  |
| Middle | 48.1 | 35 |  | 36.9 | 36.8 |  | 39.4 | 28.3 |  |
| Upper middle | 27.6 | 23.7 |  | 38.6 | 24.3 |  | 33.9 | 34.3 |  |
| Affluent | 4.5 | 10.5 |  | 5.9 | 12.0 |  | 1.2 | 1.2 |  |
| ^a^p-value based on chi-square tests for differences in the proportion responding across year  ^b^For adults aged ≥ 20, normal weight = BMI < 25 kg/m^2^; overweight = 25 kg/m^2^ ≤ BMI < 30 kg/m^2^; obese = BMI ≥ 30 kg/m^2^; in 2015 n=188 with missing BMI  ^c^2015 answer choices; 2010 answer choices: individual practice, group practice, hospital or clinic  ^d^2015 answer choices: Poor (< $25,000), lower middle ($25,000 - $49,000), middle ($50,000 - $99,000), upper middle ($100,000 - $249,000), upper (≥$250,000). 2010 answer choices: very poor-poor; poor - lower middle class; lower middle class - middle class; middle class - upper middle class; upper middle class - affluent | | | | | | | | | |
