# Supplementary material for: Changes in primary healthcare providers’ attitudes and counseling behaviors related to dietary sodium reduction, DocStyles 2010 and 2015
Source: PLoS One. 2017 May 22;12(5):e0177693. doi: 10.1371/journal.pone.0177693 (PMC5439686; doi:10.1371/journal.pone.0177693)
Supplement: S2 Table — (DOCX) [file pone.0177693.s002.docx]

| **S2 Table: Primary healthcare providers' attitudes and counseling related to dietary sodium reduction, by provider type, DocStyles 2010 and 2015** | | | | | | | | | |
| --- | --- | --- | --- | --- | --- | --- | --- | --- | --- |
|  | **Family/general practitioner** | | | **Internist** | | | **Nurse practitioner** | | |
|  | **2010** | **2015** | **P-value^a^** | **2010** | **2015** | **P-value^a^** | **2010** | **2015** | **P-value^a^** |
| **Agreement with statement "Most of my patients should reduce their sodium intake." (%)**^b^ |  |  |  |  |  |  |  |  |  |
| Strongly disagree | 0.4 | 1.5 | 0.001 | 0.2 | 0.8 | 0.01 | 0.4 | 2.8 | 0.35 |
| Disagree | 2.8 | 6.7 |  | 2.6 | 4.7 |  | 2.0 | 4.0 |  |
| Neither agree nor disagree | 12.6 | 17.9 |  | 8.5 | 13.6 |  | 12.6 | 16.7 |  |
| Agree | 54.9 | 48.8 |  | 54.9 | 50.7 |  | 57.5 | 46.2 |  |
| Strongly agree | 29.3 | 25.2 |  | 33.8 | 30.3 |  | 27.6 | 30.3 |  |
| **Which of the following types of patients do you advise to consume less salt?** |  |  |  |  |  |  |  |  |  |
| Pre-hypertensive patients | 69.2 | 68.4 | 0.78 | 65.1 | 68.2 | 0.29 | 59.5 | 70.5 | 0.009 |
| Hypertensive patients | 78.1 | 83.4 | 0.03 | 75.1 | 85.1 | <.0001 | 64.2 | 83.7 | <.0001 |
| Chronic kidney disease patients | 67.7 | 68.8 | 0.71 | 65.3 | 71.6 | 0.03 | 58.7 | 74.1 | 0.0002 |
| Diabetic patients | 45.3 | 42.6 | 0.39 | 44.3 | 50.8 | 0.04 | 38.6 | 53.8 | 0.0006 |
| Hispanic patients | 18.6 | 20.9 | 0.36 | 18.0 | 19.6 | 0.51 | 18.9 | 36.3 | <.0001 |
| African American patients | 34.5 | 41.5 | 0.02 | 33.6 | 41.5 | 0.01 | 33.1 | 50.6 | <.0001 |
| American Indian patients | 14.5 | 18.5 | 0.09 | 14.1 | 18.3 | 0.07 | 14.2 | 28.7 | <.0001 |
| Asian patients | 12.8 | 18.3 | 0.02 | 13.2 | 15.7 | 0.27 | 10.6 | 23.9 | <.0001 |
| Adults older than 40 years old | 19.1 | 25.0 | 0.03 | 19.5 | 21.3 | 0.49 | 22.4 | 34.3 | 0.003 |
| **What specific advice do you provide patients to consume less salt?** |  |  |  |  |  |  |  |  |  |
| Read nutrition labels for sodium content | 85.5 | 77.6 | 0.001 | 85.6 | 67.7 | <.0001 | 89.8 | 85.9 | 0.10 |
| Give examples of specific foods to avoid | 74.0 | 65.2 | 0.002 | 79.4 | 62.8 | <.0001 | 83.5 | 70.5 | 0.0005 |
| Remove the salt shaker from the table | 67.5 | 56.1 | 0.0002 | 67.9 | 55.1 | <.0001 | 73.6 | 61.4 | 0.003 |
| Eat less processed food | 88.5 | 76.3 | <.0001 | 82.9 | 75.1 | 0.003 | 90.2 | 88.1 | 0.45 |
| Cook with less sodium | 73.7 | 66.9 | 0.02 | 71.2 | 63.6 | 0.01 | 75.2 | 72.1 | 0.43 |
| ^a^p-value based on chi-square tests for differences in the proportion responding across year  ^b^based on Mann Whitney U test | | | | | | | | | |
